# A Rare Case of Large Osteochondral Fracture of Patella

**DOI:** 10.7759/cureus.41245

**Published:** 2023-07-01

**Authors:** Udit Agrawal, Vivek Tiwari, Rajkumar Selvanayagam

**Affiliations:** 1 Orthopaedics, All India Institute of Medical Sciences, Bhopal, IND; 2 Orthopaedics, Apollo Sage Hospital, Bhopal, IND

**Keywords:** case report, large, fracture, osteochondral, patella

## Abstract

Osteochondral fractures of the patella are relatively common pediatric knee injuries, often missed during the initial evaluation, and almost always associated with acute patella dislocations. We report the case of an adolescent patient with a very large osteochondral fracture of the patella involving almost the whole of the medial patellar facet and without concomitant dislocation of the patella. A 16-year-old adolescent presented to the emergency with pain and swelling in the left knee after sustaining a road traffic accident. On evaluation with an X-ray and a CT scan, a large osteochondral fracture of the patella was diagnosed. The fracture was treated with open reduction and internal fixation with headless compression screws after performing medial parapatellar arthrotomy. After two years, the patient recovered with a full and painless range of movement of the knee, with the complete radiological union of the fracture. This case report discusses a rare case of an adolescent with a large osteochondral fracture of the patella without concomitant patella dislocation.

## Introduction

Patella is one of the largest sesamoid bones in our body that is essential for the extensor mechanism of the knee [1]. Nearly 1% of all bone fractures comprise the osteochondral fractures of the patella [2]. These fractures occur as a result of sheer force that develops due to patellar dislocation and hitting the femoral condyle and subsequent patellar relocation. Osteochondral fractures can be associated with soft tissue injuries, including anterior cruciate ligament (ACL) tear [3]. Based on the thickness and size of the fractured subchondral bone, various modalities of treatment options are available that range from resection of fracture fragments to fixation using different methods [1,4]. In cases of grossly displaced large osteochondral fracture fragments as in this case, open reduction and internal fixation are necessary to achieve a satisfactory functional outcome [5]. The majority of principles of intraarticular fractures remain valid even for the management of large osteochondral fractures that include the reconstruction of articular congruity, restoring joint stability, achieving stable fixation, and allowing early joint motion [6]. Osteochondral fractures of the patella are often missed during the initial evaluation and are almost always associated with acute patella dislocations. We report the case of an adolescent patient with a very large osteochondral fracture of the patella involving almost the whole of the medial patellar facet and without concomitant dislocation of the patella.

## Case presentation

**Figure 1:**
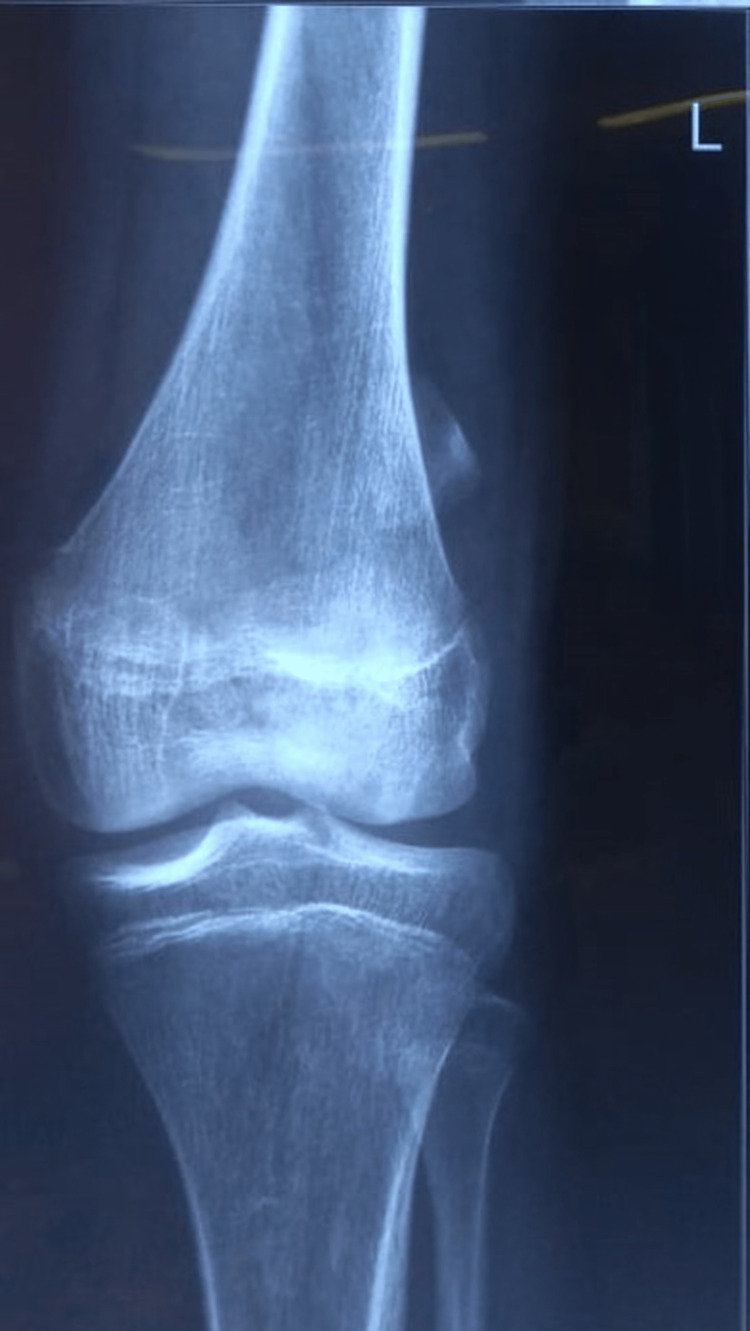
Preoperative anteroposterior roentgenogram

**Figure 2:**
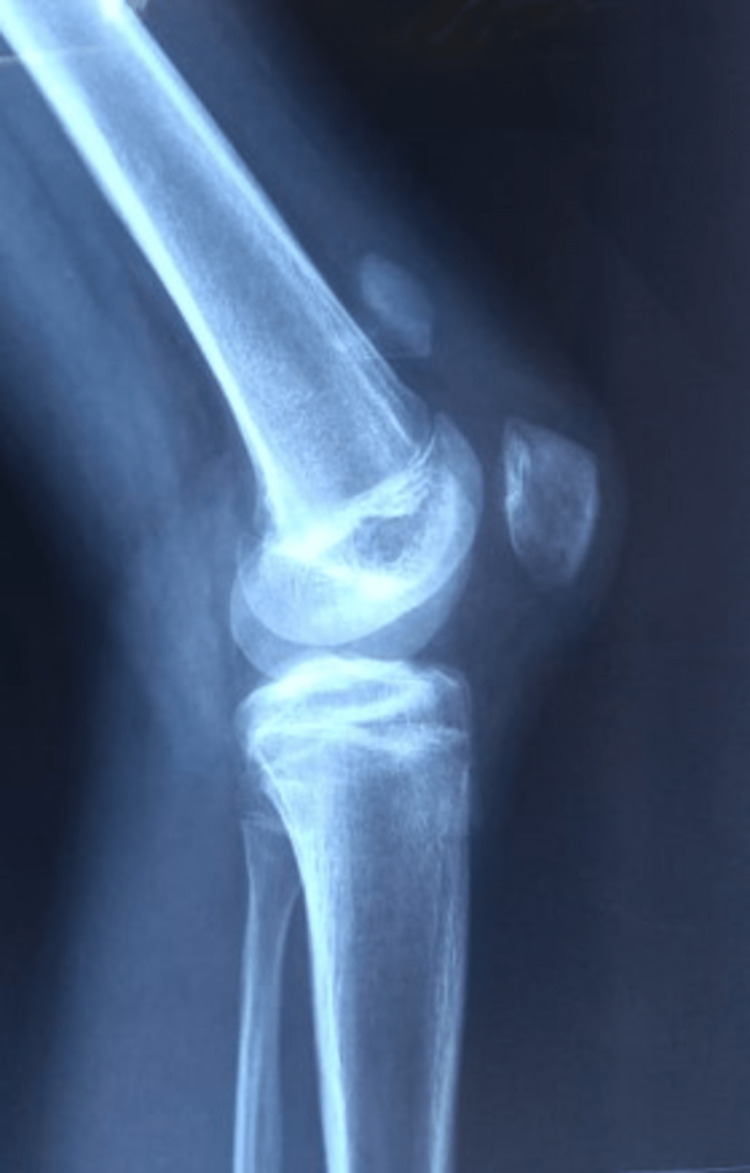
Preoperative lateral roentgenogram

A 16-year-old male patient with an alleged history of road traffic accident following which he sustained a closed injury to his left knee presented to our trauma emergency with a chief complaint of pain in his left knee. On physical examination, tenderness and minimal effusion were present over the patella. The neurovascular examination was normal. The radiological investigation was advised including an anteroposterior (AP) and lateral roentgenogram of the knee and the large displaced (6 cm) osteochondral fracture of the patella was diagnosed (Figures 1 and 2). Open reduction and internal fixation procedures were planned for the same. 

**Figure 3:**
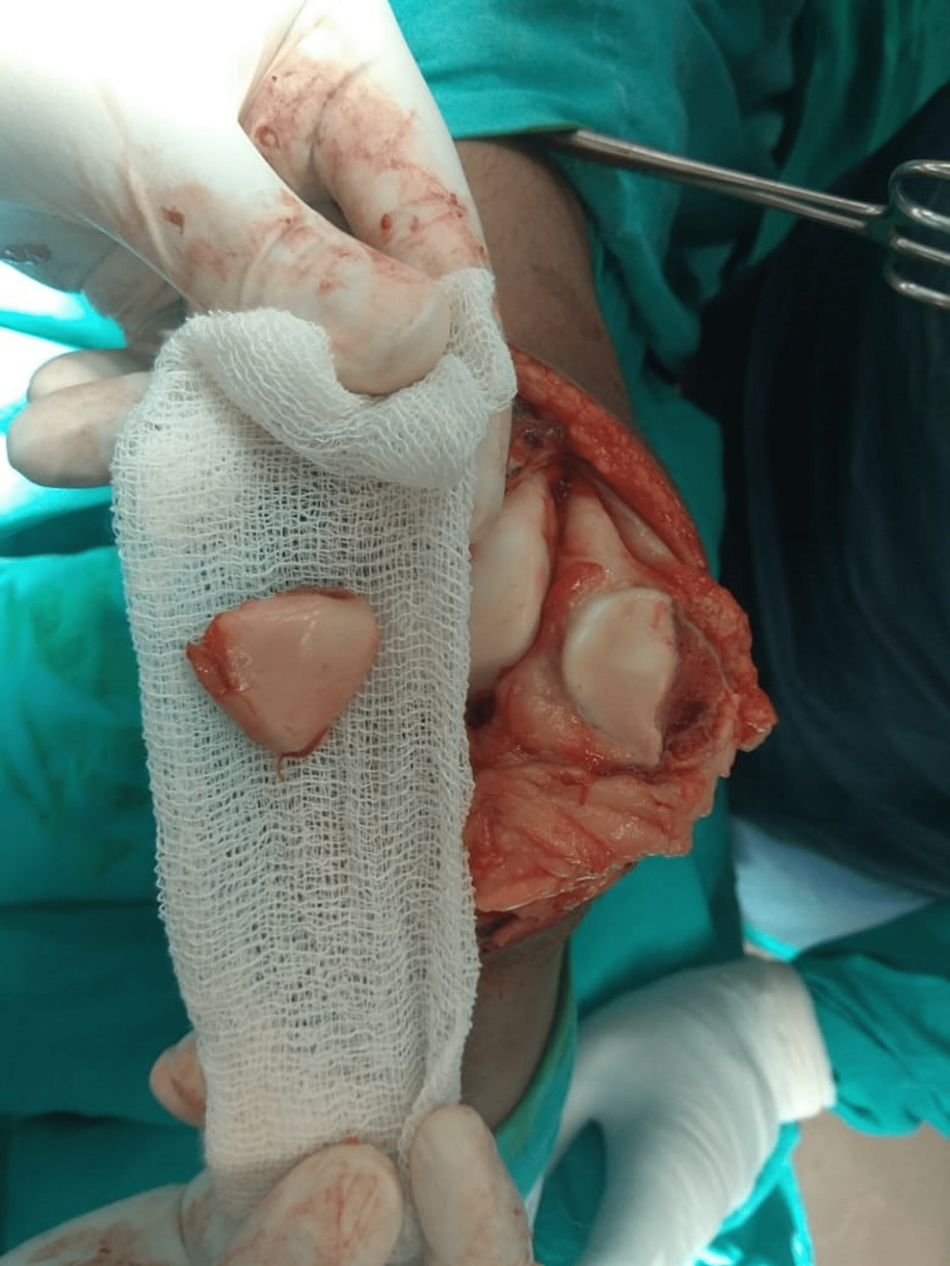
Intraoperative picture showing a large osteochondral fragment

**Figure 4:**
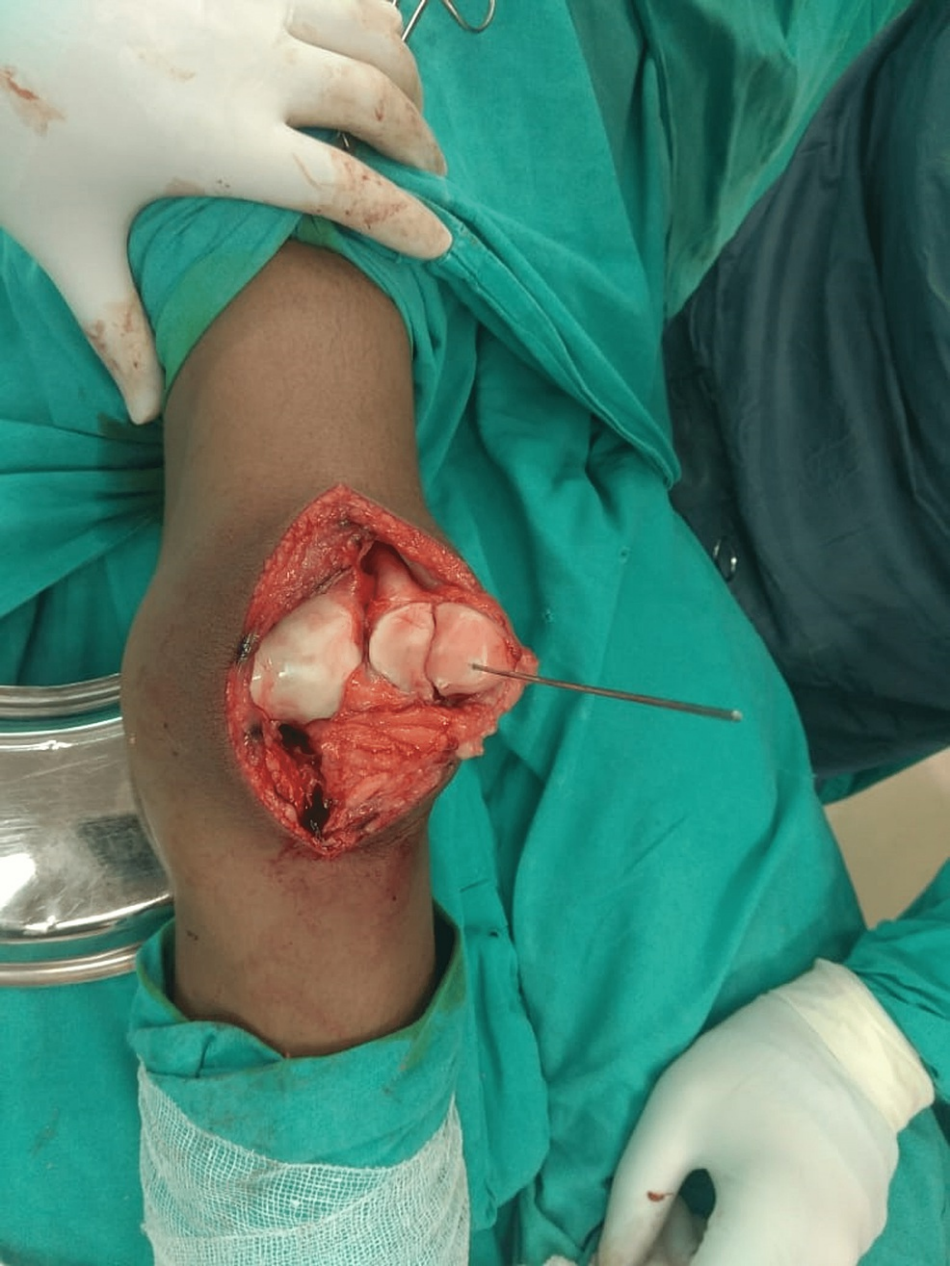
Intraoperative picture showing the provisional fixation of the osteochondral fragment

**Figure 5:**
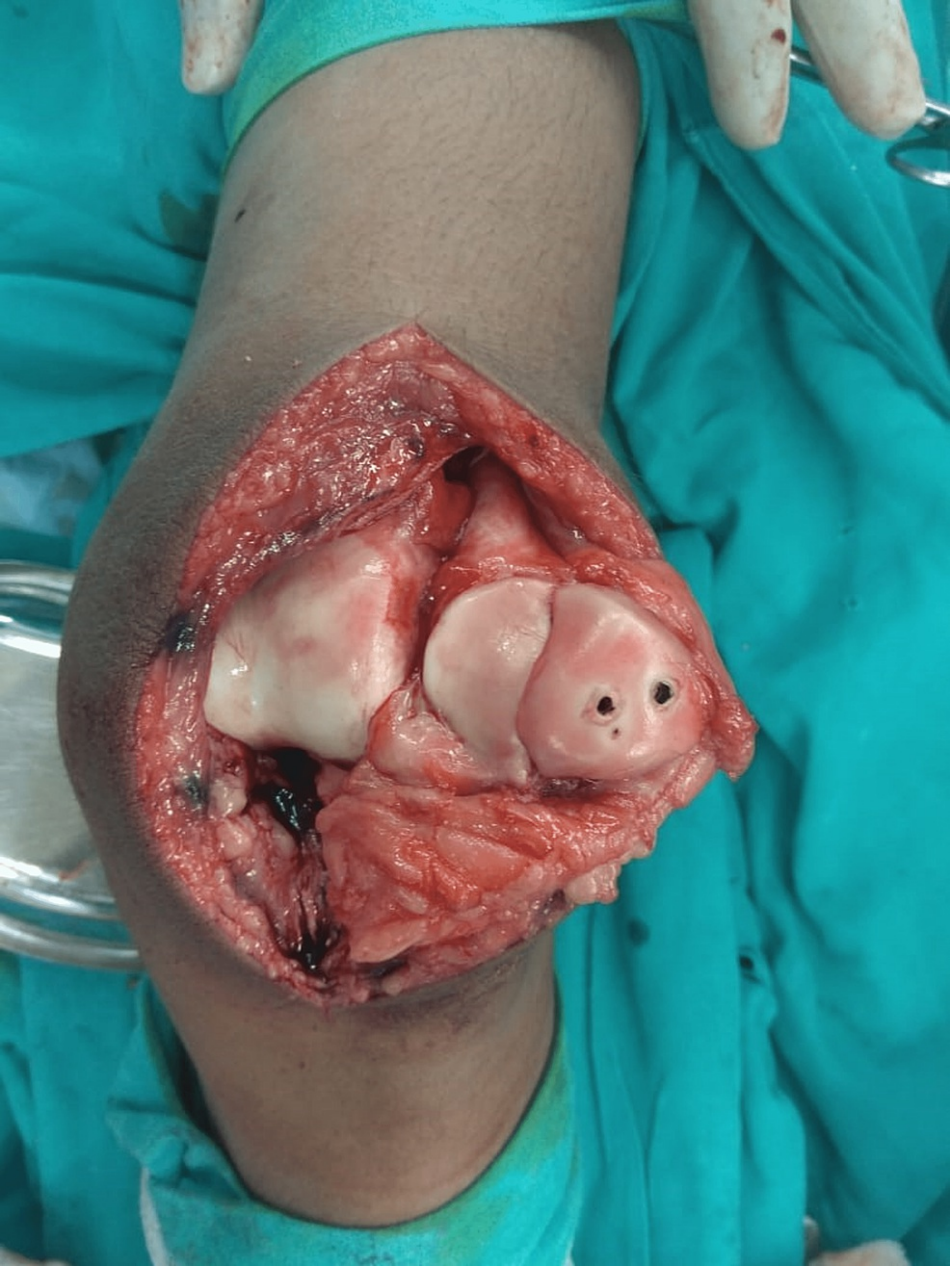
Intraoperative picture showing the definitive fixation of the osteochondral fragment using the two headless compression screws

**Figure 6:**
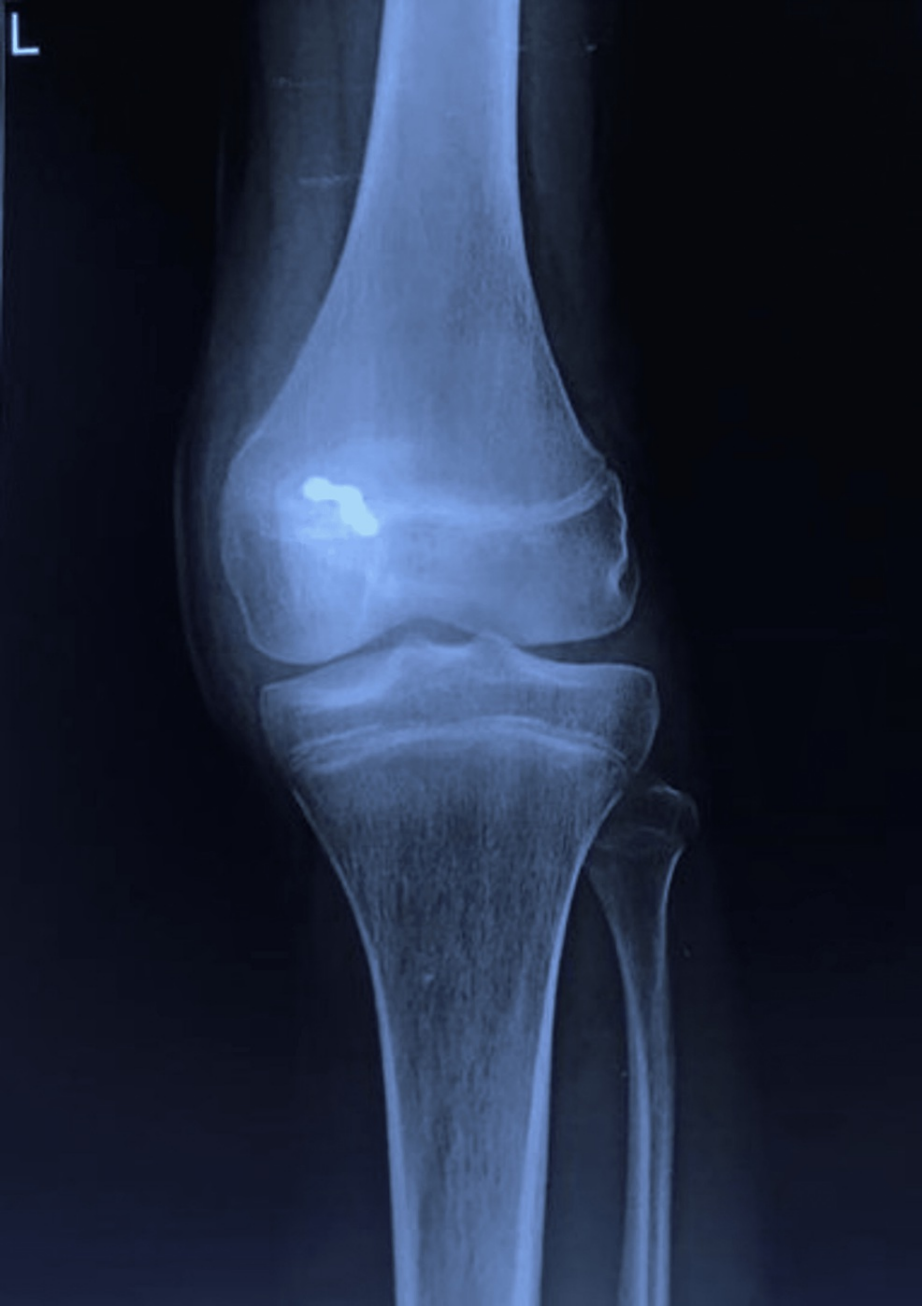
Postoperative anteroposterior roentgenogram

**Figure 7:**
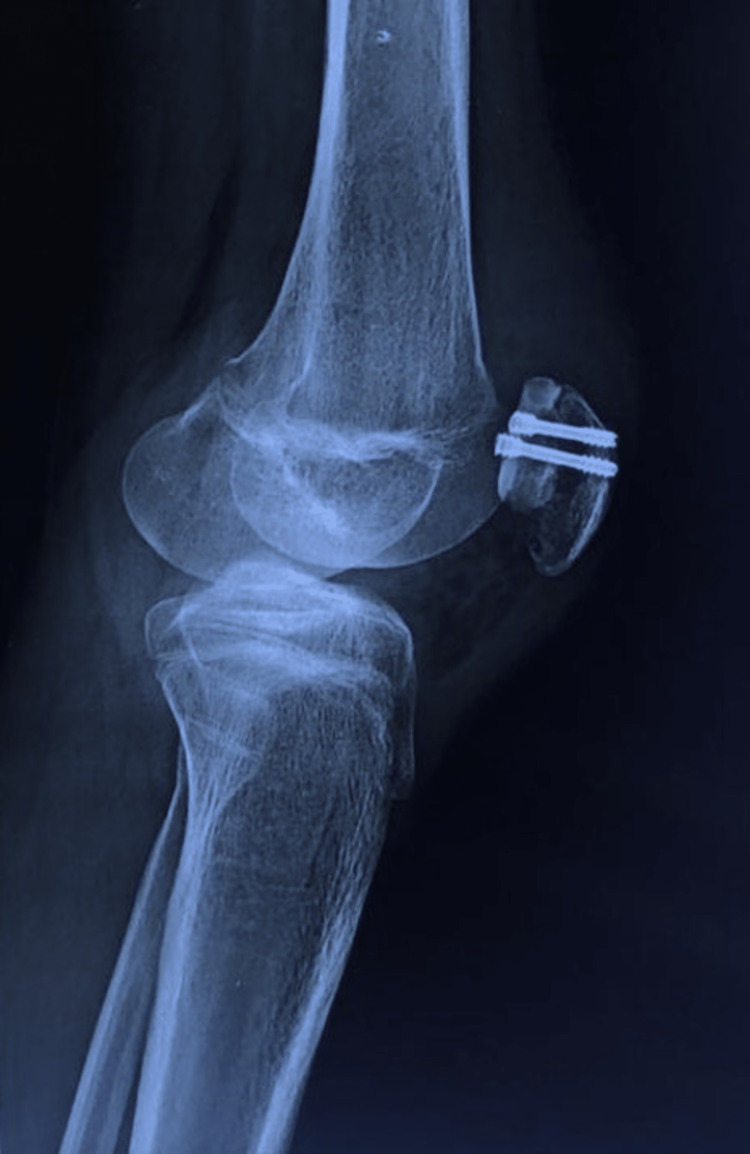
Postoperative lateral roentgenogram

**Figure 8:**
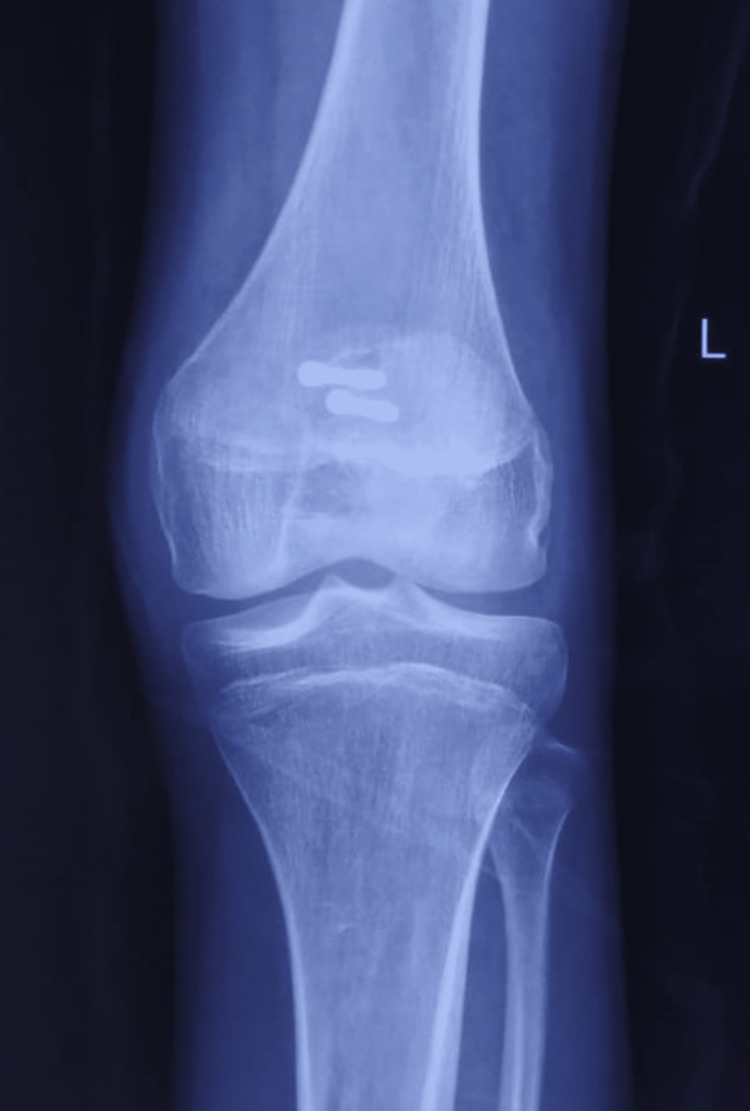
Anteroposterior roentgenogram at two-year follow-up

**Figure 9:**
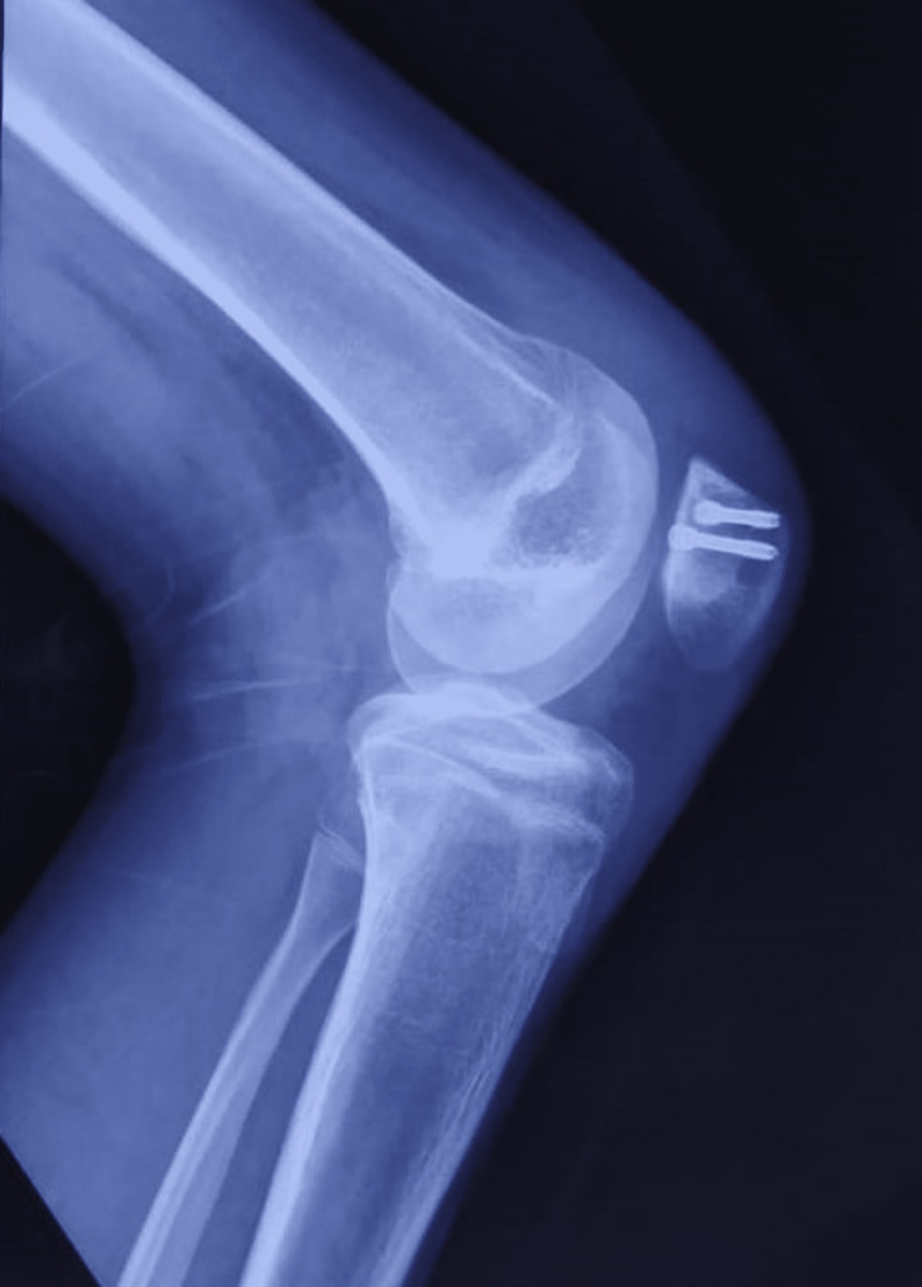
Lateral roentgenogram at two-year follow-up

**Figure 10:**
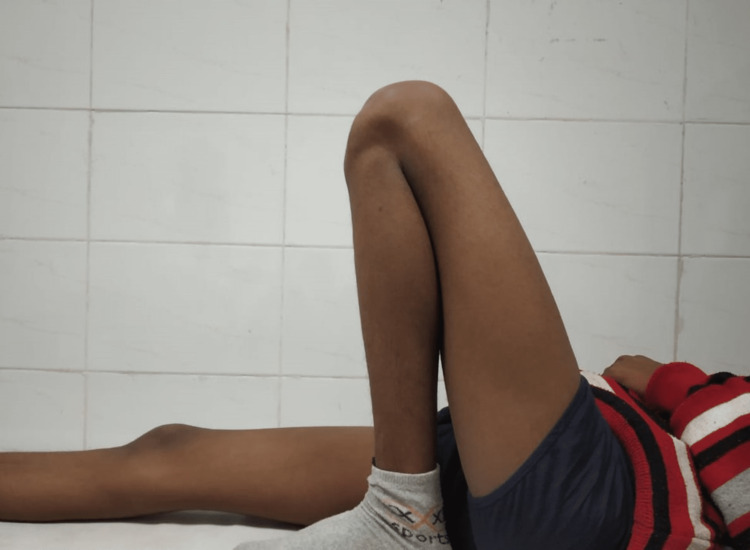
Full flexion achieved at two-year follow-up

**Figure 11:**
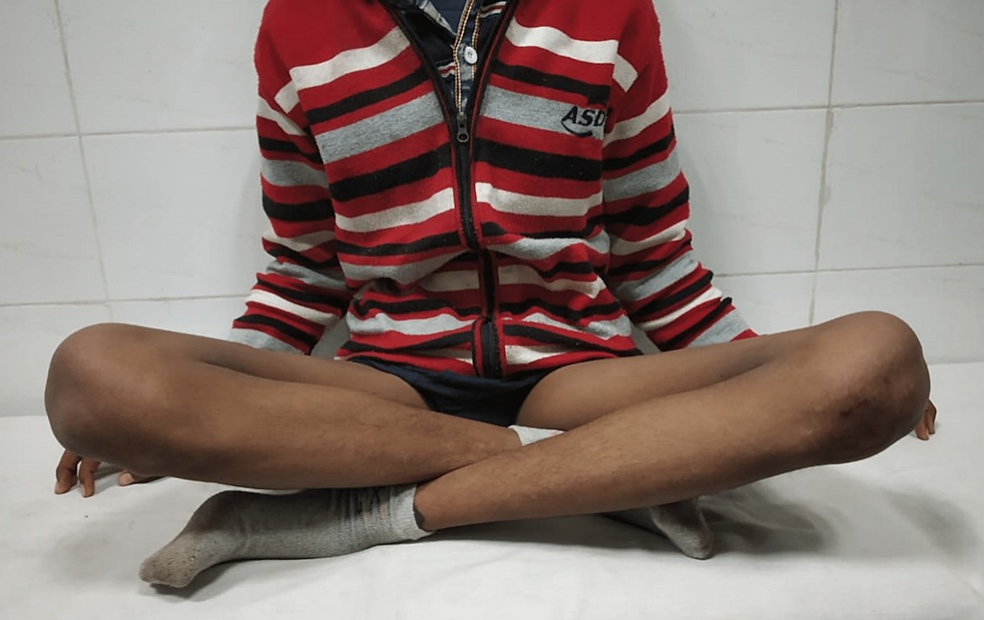
Cross-leg sitting at two-year follow-up

**Figure 12:**
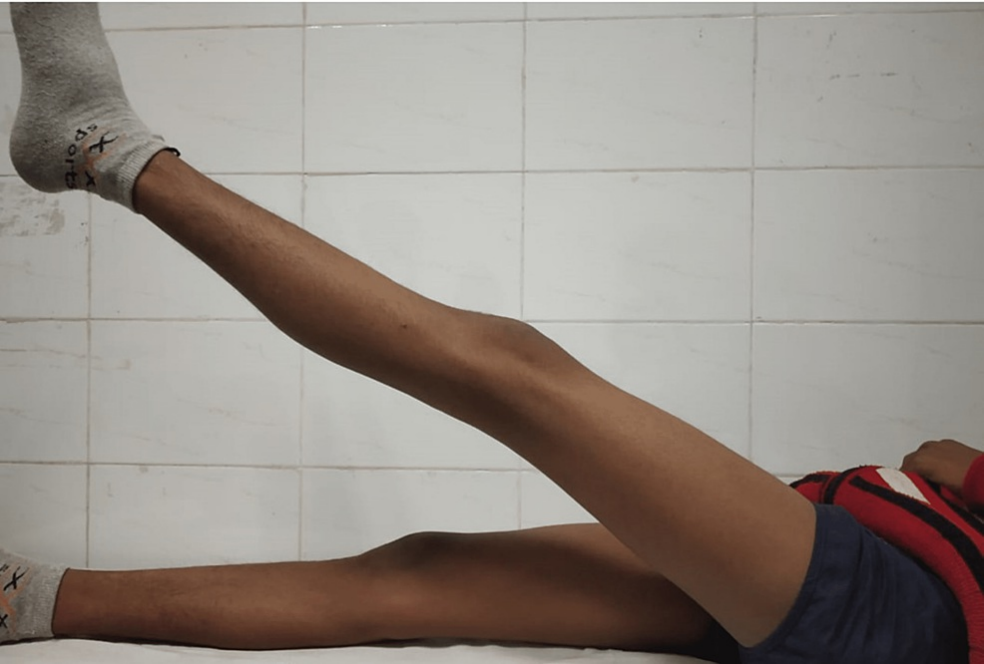
Full extension at two-year follow-up

Under spinal anesthesia, the operation was performed using a standard medial parapatellar approach. The extensor mechanism was found to be intact. A large osteochondral fracture fragment involving almost the whole of the medial patellar facet was present on assessment of the patellar joint surface (Figure 3). The fracture was reduced, and fixation was done using two full-threaded headless compression screws (Figures 4 and 5). A postoperative AP and lateral roentgenogram was done and found acceptable (Figures 6 and 7). Follow-up of the patient was done at regular intervals. At the two-year follow-up, the patient achieved satisfactory radiological (Figures 8 and 9) and functional outcomes (The knee ROM at the final follow-up was from 0° extension to 140° flexion, and he is completely pain-free.) (Figures 10, 11, and 12).

## Discussion

Traumatic osteochondral fractures of the patella are one of the most common injuries seen in the orthopedic surgery clinic and may occur at any age [7]. However, young people are more susceptible to such injury. Most of the time, AP and lateral roentgenogram views of the knee are sufficient for reaching a diagnosis as in this present scenario [2]. Such injuries are usually associated with patellar dislocation [8]. It was reported that the most common area of patellar osteochondral fracture in these patients is the medial facet of the patella [6]. The stability of the patellofemoral joint is extremely crucial to prevent early knee osteoarthritis. Niemeyer et al. suggested that any osteochondral flake fracture indicates surgical treatment with the objective of internal fixation in both pediatric and adult patients [9]. Alosaimi et al. and Bhatt et al. also reported a similar case report of osteochondral fracture of the patella not associated with patellar dislocation in the adolescent age group [7,10]. However, our patient had a very large osteochondral fracture fragment involving almost the whole of the medial patellar facet. Hence, due to the improved understanding of patellofemoral pathologies, a variety of arthroscopic and open-surgical concepts for the repair of osteochondral lesions and the restoration of joint stability have been developed [9]. Multiple treatment options are available for managing osteochondral fractures, including fixation of the displaced fragment, use of the regenerative procedure, or removing the osteochondral fragment [7]. Various studies are in favor of fixing the fragment, which seems to be a rational and ideal treatment for a large displaced osteochondral fragment [1,4,9,10]. The patient has complete and painless restoration of the knee ROM after the procedure. He was advised physiotherapy in the immediate postoperative period in the form of isometric quadriceps strengthening exercises and knee ROM exercises.

## Conclusions

This case report described a rare case of pediatric osteochondral fracture of the patella, without concomitant acute patella dislocation. The fracture was successfully managed with open reduction and internal fixation using headless compression screws. The child had an excellent outcome after two years with a complete painless knee ROM with the radiological union of the fracture.
